# 

**Published:** 1898-07-23

**Authors:** 


**Notices of Births, Deaths, and Marriages are inserted in this column at a charge of 2s. 6d. for an announcement not exceeding four lines.**

**DEATH.**

PREECE.—On July 10th, at Great Malvern, of tuberculosis, Laura Mary Louisa, daughter of Emily M. Preece, and late nurse at Plaistow Hospital.

### NOTICE TO CORRESPONDENTS.

**All MS., Letters, Books for Review, and other matters intended for the Editor should be addressed** The Editor, "The Hospital," The Hospital Building, 28 & 29, Southampton Street, Strand, W.C.

**The Editor cannot undertake to return rejected MS., even when accompanied by stamped directed envelope.**

**Notice to Advertisers and Subscribers.**

**All Advertisements, Orders for Copies of** The Hospital, **and Business Communications should be addressed to THE MANAGER, (Not to The Editor),** The Hospital, The Hospital Building, 28 & 29, Southampton Street, Strand, London, W.C.

**The Cost of Subscription, post free, is as follows:—Per week, 2½d. per quarter, 2s. 8d.; per half-year, 5s. 3d.; per annum, 10s. 6d. Foreign:—Per annum, 15s. 2d.; payable, in all cases, in advance.**

**NOTICE.—Vol. XXIII. of "The Hospital" (October, 1897, to March, 1898), in handsome cloth binding, is now on sale at the Office of this Journal, price 7s. 6d. Cases for binding the Numbers contained in this Volume, 1s. 6d. Index to Vol. XXIII., 2½d., post free.**

**The Monthly Part for August will be ready on August 2nd. Price 6d., by post 8d.**

### BOOKS RECEIVED.

SIMPKIN, MARSHALL, HAMILTON, KENT, AND CO.
"Perish the Baubles." By Frances Harriott Wood.

HENRY KIMPTON.

"The Health Resorts of Europe." By Thomas Lynn, M.D.

H. K. LEWIS.

"Practical Organic Chemistry." By Samuel Rideal, D.Sc. (Lond.)

SCIENTIFIC PRESS.

"London and Londoners." Edited by Rosalind Pritchard.

**Periodicals Received.**—Scalpel, Medical Press, Leisure Hour, Indian Medical Record, Blackwood's Magazine, Medical News, London, Clinical Supplement, Leeds Hospital Magazine, Girl's Own Paper, Boy's Own Paper, Literary Digest, Guy's Hospital Gazette, London Hospital Gazette, Columbus Medical Journal, English Illustrated Magazine, Cassell's Magazine, Sunday at Home, Love and War (Girl's Own Paper Supplement), Medical Record, Medical Temperance Review.

